# Opioid System and Epithelial–Mesenchymal Transition

**DOI:** 10.3390/ph18010120

**Published:** 2025-01-17

**Authors:** Marzena Łazarczyk, Dominik Skiba, Michel-Edwar Mickael, Kinga Jaskuła, Agata Nawrocka, Piotr Religa, Mariusz Sacharczuk

**Affiliations:** 1 Department of Experimental Genomics, Institute of Genetics and Animal Biotechnology, Polish Academy of Sciences, Postepu 36A, 05-552 Jastrzebiec, Poland; 2 Department of Medicine, Karolinska Institute, 171 77 Solna, Sweden; 3 Department of Pharmacodynamics, Faculty of Pharmacy, Medical University of Warsaw, Banacha 1B, 02-091 Warsaw, Poland

**Keywords:** epithelial to mesenchymal transition, opioid, E-cadherin, N-cadherin, vimentin

## Abstract

Opioids are a challenging class of drugs due to their dual role. They alleviate pain, but also pose a risk of dependency, or trigger constipation, particularly in cancer patients, who require the more potent painkillers in more advanced stages of the disease, closely linked to pain resulting from general inflammation, bone metastases, and primary or secondary tumour outgrowth-related nerve damage. Clinicians’ vigilance considering treatment with opioids is necessary, bearing in mind extensive data accumulated over decades that have reported the contribution of opioids to immunosuppression, tumour progression, or impaired tissue regeneration, either following opioid use during surgical tumour resection and post-surgical pain treatment, or as a result of other diseases like diabetes, where chronic wounds healing constitutes a challenge. During last few years, an increasing trend for seeking relationships between opioids and epithelial–mesenchymal transition (EMT) in cancer research can be observed. Transiently lasting EMT is desirable during wound healing, but in cancer, or vital organ fibrogenesis, EMT appears to be an obstacle to overcome, forcing to adjust treatment strategies that would reduce the risk for worsening of the disease outcome and patient prognosis. The same opioid may demonstrate promoting or inhibitory effect on EMT, dependently on various conditions in particular clinical cases. We have summarized current findings on this issue to uncover some rules that govern opioid-mediated EMT induction or repression; however, many aspects still remain to be elucidated.

## 1. Introduction

The epithelial–mesenchymal transition concerns changes in expression of crucial, structural molecules that provide spatial organization, both intracellularly and between cells within the tissue. During EMT, normal epithelial cells, sealed between themselves and at the site of contact with basement membrane, experience partial, or complete loss of mechanical and functional communication. Several types of junctions have been identified in epithelial tissues, e.g., tight junctions, adherens junctions, gap junctions, desmosomes, and hemidesmosomes [1,2,3,4]. All of these junctions are composed of specific proteins wherein changed expression is usually observed in EMT. The first type of connections formed closed to the apical area of the adjacent epithelial cells consists of transmembrane claudins, occludins, and the adaptor proteins zonula occludens (ZO-1 and ZO-2). ZOs are linked with the intracellular actin cytoskeleton. In the arrangement of adherens junctions, they locate laterally and the process involves transmembrane E-cadherins, and adaptor proteins: β-catenin and α-catenin, also forming bonds with actin filaments immersed in the cytoplasm. The second type of junctions, placed in multiple sites along the lateral cell membrane, are desmosomes, built of transmembrane proteins (desmocollins and desmogleins) and adaptor proteins (plakoglobins, plakophilins, and desmoplakins), which tie to cytosolic cytokeratins. The third type are gap junctions composed of connexins, being communication channels for mutual exchange of small molecules (e.g., ions) between cells. The fourth type are hemidesmosomes, located on the basal cell surface, and they align the cell to basal lamina by transmembrane integrins. Among these structural elements, several are often determined in studies both on EMT and mesenchymal–epithelial transition (MET), including claudins, cadherins, vimentin, and ZO-1/2. Relaxation of the external sites of contact, which hold adjacent cells together, causes not only gaining the ability to move from current position in the tissue but also allows marked cytoskeletal rearrangements within the cells, involving actin microfilaments, intermediate filaments, and microtubules (composed of tubulin). Some of the intermediate filaments, such as cytokeratins, specific for epithelial cells, or vimentin, typically expressed by mesenchymal cells [5,6], are frequently being objects in research focused on EMT. Obviously, besides genes encoding structural cell proteins, hundreds of other mediators are involved in EMT regulation [7]. The consequences of EMT-driven loss of cell–cell interactions can be invasion (degradation of surrounding extracellular matrix; ECM) by secreted metalloproteinases (MMPs) or the migration of circulating tumour cells (CTCs) through bloodstream to the distant tissues and organs, which can cause metastases formation [8]. The most-known and widely studied powerful inducer of EMT is transforming growth factor β (TGF-β), with its three isoforms TGF-β1, TGF-β2, and TGF-β3. Almost all normal cells [9], as well as cancer cells, retain the ability to secrete this cytokine, which physiologically is accumulated in the ECM in the latent state, being liberated upon activation signalling and cleavage of some dormancy-maintaining protein domains. Signal transduction factors that stimulate TGF-β include integrins, pH change, reactive oxygen species, and proteases. The canonical TGF-β-mediated signalling pathway involves SMAD proteins, while non-canonical TGF-β-governed pathways engage kinases and transcription factors such as extracellular signal-regulated protein kinase (ERK), phosphatidylinositol 3-kinase (PI3K)/AKT, p38 mitogen-activated protein kinase (MAPK), c-Jun N-terminal kinase (JNK), Janus kinase (JAK), signal transducer and activator of transcription (STAT), or nuclear factor κB (NF-κB) [10]. The human recombinant TGF-β1 protein added to the culture of human liver carcinoma HepG2 cells at the concentration of 10 ng/mL with daily media exchange, along with everyday TGF-β1 supplementation, triggers appearing predominantly detached, rounded cell clusters after 3–4 days and almost complete loss of cell adherence to the dish surface up to 7 days with no easily noticeable, apparent changes in morphology before detachment, when compared to the non-treated culture (our unpublished observations). As we observed, it is difficult to capture the moment of the transition from the epithelial-like to the elongated shape of the HepG2 cells before they become rounded and start to swim freely as sphere-shaped objects or clumps in the medium fluid, and the time for EMT markers determination of cultured HepG2 cells without substantial cell loss due to detachment elapses after 72 h. Other widely described molecules with the ability to determine the cell fate towards mesenchymal de-differentiation are interleukin 6 (IL-6) [11,12] or growth factors, such as epidermal growth factor (EGF), fibroblast growth factor (FGF), and insulin-like growth factor (IGF) [13,14,15,16,17,18], used in experimental studies on EMT. In cancer, by definition, EMT refers to the transition process and, therefore, to the lesions of epithelial origin; however, EMT-like changes, reflected by expression of some markers relevant to EMT, can be found in non-epithelial cells such as fibroblasts (mesenchymal origin) or endothelial cells [19], as well as in non-epithelial malignancies [20,21,22,23,24]. Furthermore, reaching a conclusion on EMT may be difficult following determination of only few EMT markers. For example, ZO-1 upregulation is usually linked with decreased cancer progression, EMT inhibition, or even a reversed process called MET, when other hallmarks are also present and support epithelial phenotype restoration. Surprisingly, in melanoma, being a malignancy of non-epithelial origin, ZO-1 is linked with N-cadherin in adherens junctions between melanoma cells themselves and at contact sites with surrounding fibroblasts, while ZO-1 knockdown switches melanoma cell morphology from dendritic to a more rounded shape [25]. Moreover, although cancer cells acquire an invasive phenotype after EMT, the detectability of some EMT markers in metastatic tissues may be misleading, especially when assessing the stage of cancer. During EMT, β-catenin splits from E-cadherin and translocates from the cytoplasm to the nucleus, where it activates EMT-related transcription factors. Thus, a reduction in β-catenin–E-cadherin complexes is linked with increased primary tumour invasiveness; however, in metastatic tissues and, therefore, in advanced stages of cancer, re-expression of these proteins can be found [26], perhaps indicating metastatic cell colonization and restoration of relevant epithelial markers expression, facilitating settlement in a new place.

In the EMT, expression of at least one of the crucial transcription factors—zinc finger E-box binding homeobox (ZEB), snail family transcriptional repressor 1 (SNAIL), snail family transcriptional repressor 2 (SLUG), or a twist family of basic helix-loop-helix (bHLH) (TWIST)—is usually detected in cells undergoing the transition, both in normal process and malignant transformation [27,28,29,30,31,32]. These factors are repressors of transcription of the genes encoding proteins, which are responsible for epithelial characteristics of the cells. Changes in cell–cell junctions and cytoskeletal rearrangement may be incomplete and result in migration of cells collectively, in a form of clumps. Thus, it is not uncommon that EMT-driven cells display simultaneous expression of epithelial (e.g., E-cadherin) and mesenchymal (N-cadherin, vimentin) markers [33]. Although acquiring stemness or resistance to chemotherapy by cancer cells may be preceded by changes in some EMT markers, recent consensus advises caution in associating such acquired cellular competences with canonical EMT [33].

Numerous molecules that participate in EMT may be considered as potential targets for the development of specific, anti-invasive drugs dedicated to the treatment of cancer. An obvious limitation is the pleiotropic effect of these molecular targets in the regulation of more than one signalling pathway, of which some may be necessary for maintaining normal homeostasis. Indeed, some propositions of such EMT-attenuated drugs have emerged [34,35,36].

Over the last few decades, researchers’ efforts have allowed to observe and experimentally confirm adverse effect of opioids on cancer in patients undergoing surgical resection of tumour, receiving opioids for post-surgery or cancer-related pain, particularly in the advanced stages of the disease. Use of these specific group of painkillers may contribute to the accelerated disease progression. It is commonly accepted that surgery, per se, is the immunosuppressive event, being a burden for the immune system, already weakened by the endless facing of cancer antigens that reproduce themselves concomitantly with cancer cell proliferation. Hence, constant efforts are undertaken to limit opioid use to the clinically justified cases, when patients experience severe pain that is hard to endure. Opioids, both essential, endogenously produced, and synthetically derived are used in relevant, clinical conditions, and they act through µ-, δ-, and κ-opioid receptor types (further referred to as MOR, DOR, and KOR, respectively). The opioid system has been evidenced to modulate several molecular processes, which are pivotal for tumour progression, such as cell growth, survival, proliferation, invasion, migration, and angiogenesis. The regulatory activity of the opioid system may occur at several levels and concern not only cancer cells but also the tumour microenvironment, including immune cells infiltrating the lesion, tumour stroma, or endothelial cells of tumour vasculature. Multiple opioid-mediated mechanisms of cancer development, progression, and metastasis have been defined. One of them is the effect on EMT-related markers, which has recently attracted increasing attention. We have reviewed current research results on the effect of opioid substances on EMT in pathological conditions such as cancer and tissue fibrosis, as well as in a normal wound-healing process. To collect relevant publications, we have used PubMed and ScienceDirect databases, using entries: “opioid” AND “epithelial–mesenchymal transition” for seeking records containing these two phrases at once in all fields (title, abstract, and main text). Available data, linking EMT with opioids, largely originate from cancer research. To avoid repetitions in the text, each time when “essential/classical EMT markers, including” is mentioned, it denotes that the authors of a particular analyzed paper on opioids and EMT have established opioid-induced changes in E-cadherin, N-cadherin, and vimentin, if not specified otherwise. Word “including” means additional, determined proteins related to EMT, apart from checked expression of the three, mentioned signatures.

## 2. Opioids, EMT, and Cancer

The latest global statistics on cancer incidence throughout human population from all continents are now available for year 2022 with an estimated nearly 20 million new cases of cancer indicated in 186 countries. Lung cancer ranks first, with the highest number of new diagnoses, followed by malignancies of the breast, colorectum, prostate, etc. [37]. Therefore, in such order we have decided to present findings on relationships between EMT and opioids in particular cancer types. The list of EMT signatures that have been determined in the studies involving opioids is presented in Table 1.

### 2.1. Lung Cancer

Expression levels and distribution of opioid receptors in a tumour compared to healthy tissues often reflect the necessity of their presence or absence for the malignant transformation and lesion expansion. Increased MOR staining intensity in clinical lung cancer samples compared to adjacent healthy tissues or metastatic samples indicates the involvement of MOR in tumour development [40].

In human lung cancer H358 cells, MOR ligands as morphine, [D-Ala^2^, MePhe^4^, Gly(ol)^5^]enkephalin (DAMGO), or fentanyl were proved to upregulate vimentin, SNAIL, and SLUG expression that entailed concomitant downregulation of claudin-1 and ZO-1 levels in a concentration-dependent manner (Table 2). A similar effect on EMT-related marker expression pattern was achieved following transduction of these cells with a human *OPRM1* gene-containing vector, which resulted in MOR overexpression. MOR-mediated signalling pathway suppression by the encoding gene silencing or pretreatment with antagonists (naloxone or peripherally acting methylnaloxone) reversed the initially uncovered EMT-stimulatory effect of the opioid agonists. The use of several inhibitors allowed for determination of additional molecular players involving in this activated, MOR-mediated pathway, such as SRC, PI3K, AKT, STAT3, or growth factor receptor-bound protein 2 (GRB2) [41].

On the contrary, another MOR agonist, sufentanil, being a derivative of potent opioid agonist fentanyl, decreased invasion and migration of human H460 and H1299 lung cancer cells, which was reflected at the molecular level by upregulation of ZO-1 typically present in tight junctions, changes in cadherins and vimentin levels specific for EMT withdrawal, and downregulation of WNT signalling pathway molecules: β-catenin, MYC, and MMPs [42], (Table 2).

Opioid growth factor receptors (OGFRs) exhibit no significant homology to classical opioid receptors and are associated with nuclear membrane [43]. Their essential ligand is methionine-enkephalin (MENK), also called opioid growth factor (OGF); however, MENK also displays binding affinity to DORs and MORs [44]. MENK has been shown to change EMT markers expression towards a less invasive phenotype, including downregulation of MMP2 in human lung cancer A549 and H1975 cells via interaction with OGFR, followed by migration inhibition, while knockdown of *OGFR* and exposure to MENK resulted in a reversed EMT marker expression pattern, possibly through an alternative, classical opioid receptor-mediated pathways [45], (Table 2). Interestingly, in animal study, tumours from A549 cancer cell-bearing mice administered with MENK demonstrated significantly lower interleukin 10 (IL-10) and TGF-β1 levels and contained more M1 macrophages than the pro-tumorigenic M2 type [46], when compared to tumour tissues from non-treated control animals [45], similarly as it was shown in murine Lewis lung cancer-bearing mice treated with MENK [47]. Probable activation of other opioid receptors by MENK, in the absence of OGFR in A549 cells resulting in increased invasiveness, supports data presented in another report on the same lung cancer A549 cell line (Table 2). Upon MOR stimulation by morphine, E-cadherin levels were decreased both in A549 and Lewis lung cancer cells and the effect, associated with activated MOR-mediated increase in phosphorylation of downstream kinases AKT, PI3K, and mammalian target of rapamycin (mTOR), was reversed by naloxone [48].

### 2.2. Breast Cancer

Breast cancer is a type of malignancy ranked on the second position, considering the worldwide incidence number in 2022. In the research conducted on breast tumours, it has been proven that histologically normal tissue adjacent the visible margin of the lesion at a 1 cm distance displayed higher levels of gene transcripts, such as *IGF1*, platelet-derived growth factor receptor β (*PDGFRB*), *TGF-β3*, *SNAIL*, or vimentin, than tissue surrounding the tumours at the distance of 5 cm, which suggested that EMT-related events occur even in a seemingly healthy, neighbouring area [49].

The use of DOR agonists may cause various effects on invasive properties of cancer cells, and it has been documented that they depend, among others, on the level of DOR expression [50]. In a recent study, [D-Ala^2^, D-Leu^5^]-enkephalin (DADLE) not only enhanced migration of DOR-expressing human MCF-7, murine T47D (epithelial origin), and human MDA-MB-231 (mesenchymal origin) breast cancer cells, which was reversible upon exposure to naltrindole, but also upregulated SNAIL in all cell types [51]. However, increased TWIST and downregulated E-cadherin were observed following DADLE treatment of only cell lines of epithelial origin. Interestingly, both in naïve and treated MDA-MB-231 cells, E-cadherin was undetectable. Furthermore, in all cell types DADLE-activated DORs stimulated STAT3, a potent EMT inducer controlling crucial transcription factors during the transition [51], to a similar extent as IL-6 can do [52]. Strikingly, DADLE only had an influence on breast cancer cell migration, but not proliferation; however, such a dichotomy phenomenon was recognized in the past [53,54]. Consistently, an EMT-promoting effect was seen with a MOR agonist, tramadol, administered to an MDA-MB-231 cell culture, resulting in increased TGF-β and α-smooth muscle actin (α-SMA) along with a concomitant decrease in E-cadherin and collagen I levels and elevated expression of N-cadherin, vimentin, SNAIL, and SLUG. Interestingly, hypoxic conditions abolished these effects. Similar results were obtained in tramadol-treated MCF-7 cells (Table 2); however, the authors did not detect vimentin in this type of breast cancer [55]. On the contrary, in rat models of chemically induced breast cancer and an intravenously triggered metastatic form of the disease following breast cancer cell infusion directly to the circulation system, animals with β-endorphin-releasing neurons transplanted into the hypothalamus had reduced levels of tumour necrosis factor α (Tnf-α) and Nf-κB, as well as Snail, Slug, and Twist, with a concomitant decline in N-cadherin and increased level of E-cadherin in tumours excised from mammary glands, as compared to animals with control transplants (Table 2). Simultaneously, intravenous delivery of breast cancer cells as “metastatic seeds” failed to support metastases formation in the lungs of β-endorphin neuron-grafted rats, which was reversed by naloxone. The beneficial, therapeutic effect of β-endorphin resulted also from its influence on stress hormone-mediated, tumour-promoting pathways [56]. Another opioid agonist, nalbuphine, displaying affinity to MOR and KOR [57] when added to the human breast cancer cell cultures or administered to breast tumour-xenografted mice, resulting in decreased expression of vimentin, N-cadherin, and SNAIL1 but increased E-cadherin both at RNA and protein levels via repression of AKT-NFκB phosphorylation; therefore, this occurred through the inhibition of a powerful mitogenic and pro-migratory signalling pathway [58] (Table 2). Use of sufentanil attenuated the invasiveness of breast cancer MDA-MB-231 cells via inhibition of NF-κB and NF-κB inhibitor α (IKBα) phosphorylation without changing levels of these factors [59] (Table 2). In human epidermal growth factor receptor (HER)-positive human breast cancer BT474 cells, sufentanil, combined with parecoxib, affected essential EMT markers, including a decrease in SNAIL, pushing forward the epithelial phenotype [60] (Table 2). An approach to target more than one receptor type in EMT modulation, namely MOR and β2 adrenergic receptors (β2ARs), was followed in a study on rat and murine breast cancer cells (Table 2). When challenged to these receptors’ antagonists: naltrexone and propranolol, respectively, the combination of the two had the greatest and desirable effect on EMT-related markers as compared to single treatment with either ligand. Similarly, in tumours of MDA-MB-231 cell-inoculated rats, the most beneficial effect on epithelial and mesenchymal markers expression, including reduction in SNAIL, SLUG, and TWIST, has been achieved when a mixture of both mentioned drugs was used [61]. Since stress and stress-associated hormones have promoting influence on cancer (e.g., adrenaline promotes pancreatic cancer cell EMT and migration via β2 adrenergic receptor activation [62]), attempts to include several strategies and stress hormone-related targets at once seems to be promising, also in research on EMT [56]. Another MOR activator, oxycodone, revealed its favourable impact on EMT-related markers in human breast cancer SKBR3 cells; however, only E- and N-cadherin levels were determined and found to be upregulated and downregulated, respectively (Table 2). Oxycodone decreased AKT and mTOR kinases activation in these cells [63]. Apart from DORs or MORs, KORs are also the entrance for target-specific modulation of breast cancer progression involving EMT-related proteins. Evidence on the involvement of KOR in breast cancer EMT provided study on human MDA-MB-231 and MCF-7 cells with opioid receptor κ gene (*OPRK1*) siRNA-downregulated KOR expression, where more pronounced changes were observed in the KOR-deficient breast cancer cells with increased E-cadherin but decreased N-cadherin, SNAIL, vimentin, and phosphorylated AKT kinase at protein levels in comparison to control cell cultures [64].

During seeking for potential therapeutic substances against cancer, in silico analyses are widely used. Virtual screening of FDA-approved drugs targeting insulin receptor (IR), integrin β1 (ITGB1), and a cluster of differentiation 36 (CD36) molecules involved both in metabolism and EMT, that would be promising in breast cancer therapy, pointed out few substances, with the opioid naldemedine among them [65]. The drug is a peripherally acting antagonist of MOR and DOR, being prescribed for cancer patients with opioid-induced constipation [66]. So far, no data on the naldemedine effect on EMT mediators were reported, and the findings from such simulated screening [65] require further in vitro/in vivo verification. However, there are some premises that naldemedine may inhibit cancer progression. In mice injected sc. with Lewis lung carcinoma or B16 melanoma, this opioid antagonist suppressed tumour growth. The effect was reproduced with the use of another MOR antagonist methylnaltrexone, which also does not cross the blood–brain barrier; however, another peripherally acting opioid antagonist, loperamide, demonstrated tumour-promoting properties in this study [67]. Whether the effect of naldemedine on cancer is mediated by changes in EMT markers, as suggested by drug repurposing analyses, remains an open question.

One of the discrepancies between studies on breast cancer, including EMT, concerns differently described MDA-MB-231 cell lines, reported as either of epithelial phenotype (with detectable E-cadherin) or mesenchymal origin. These inconsistencies remain to be clarified, since in studies focusing on EMT, real transition and, thus, reprogramming from an epithelial to mesenchymal phenotype, or EMT-like processes, are recommended to be clearly distinguished [33].

### 2.3. Colorectal Cancer

Cancer of the colorectum is on the third position of incidence frequency among malignancies in global statistics. Encouraging results targeting opioid receptors and EMT-mediated mechanisms in progression of this type of cancer have been obtained in experiments on human colorectal carcinoma HCT116 cells. MOR silencing led to downregulation of β-catenin and TWIST, simultaneously shifting cadherins and vimentin expression levels towards more epithelial “direction”. A specific AKT kinase activator restored the EMT phenotype in these MOR-lacking cells, revealing PI3K/AKT mediators as crucial downstream players in an EMT-aiming pathway governed by stimulated MOR [68].

### 2.4. Prostate Cancer

Drug repurposing libraries are useful tools for screening available and usually approved therapeutic agents when searching new indications for old substances. Recently, among over a thousand pharmacologically active compounds, only three substances, including one opioid, namely naltrexone hydrochloride, have been appointed as promising agents for EMT suppression. Unique opioid antagonist properties relied on capability to induce substantial increase in E-cadherin level, particularly in the cell junction areas of human prostate PC3 cancer cells. Screening was based on the evaluation of chemicals’ capacity to switch FGFR2 splicing from exon 9 (with the IIIc protein isoform as a product, linked with an increased invasive phenotype in prostate cancer) to exon 8 (yielding in an IIIb FGFR2 isoform, associated with decreased malignancy). Since normal epithelial tissues display higher expression of the IIIb protein variant than IIIc; while IIIc is preferentially expressed in mesenchymal cells, the actual capability of naltrexone to stop EMT-associated events has been revealed [69]. Further exploration of naltrexone as adds-on in oncological therapy is justified, since prostate cancer is the fourth most common type of malignancy worldwide.

### 2.5. Liver Cancer

Analysis of human hepatocellular carcinoma (HCC) tissues in relation to patient overall survival showed poorer prognosis for those having tumours expressing MORs and vimentin or MOR with concomitant absence of E-cadherins. Furthermore, HCC MHCC-97H cells with silenced MOR expression demonstrated downregulation of vimentin, N-cadherin, TWIST, and SNAIL, but upregulation of E-cadherin, while another HCC cell line, HepG2, engineered to overexpress MORs, displayed the opposite expression pattern of these EMT-related proteins [70]. Further studies are required to confirm MOR usefulness as a therapeutic target in HCC; however, preliminary data seem to be encouraging for development of therapeutic MOR inhibitors aiming to inactivate downstream EMT molecular signatures.

### 2.6. Cervical Cancer

GLOBOCAN 2022 statistics recorded almost 350,000 deaths from cancer of cervix uteri among the female world population. A new direction in developing efficient medicines for the disease perhaps would foreshadow recent research findings. In a study on human cervical cancer HeLA cells, either treatment with a low dose of naltrexone (NTX) or knockdown of the OGFRs combined with low dose NTX administration affected significantly E-/N-cadherin, vimentin, and SNAIL expression, resulting in restoration of a more epithelial and less invasive phenotype of cancer cells (Table 2). However, the effect was more pronounced upon NTX monotherapy, with simultaneous maintenance of undisturbed functionality of OGFRs. In addition, NTX diminished the number of tumour-associates macrophages (TAMs), mostly of the M2-polarized subtype in tumours of HeLa cell-inoculated mice [71].

### 2.7. Bladder Cancer

Bladder cancer is on the next position, after cervical cancer, considering worldwide incidence. Although opioid system antagonists have been mostly used in studies on EMT to antagonize the effect triggered by prior agonist use, naltrexone alone added to bladder cancer T24 and MB49 cell cultures revealed an EMT-promoting effect, reflected by a changed cell morphology into a spindle shape and altered expression of classical EMT markers, including elevation in SLUG and SNAIL levels (Table 2). Moreover, activation of PI3K and AKT were uncovered in NTX-treated cells following specific PI3K inhibitor-blocked phosphorylation of the kinase that resulted in suppression of changes in expression of the transition markers [72]. Interestingly, another experiment on cultures of identical cell lines exposed to morphine, therefore, upon only MOR activation, also evidenced a stimulatory effect on EMT with PI3K/AKT involvement and a significant increase only in SLUG levels, but not SNAIL, SNAI3, ZEB1/2, neurogenic locus notch homolog protein 1 (NOTCH1), TWIST, or WNT1 (Table 2). SLUG or MOR silencing abolished EMT-promoting effect of morphine [73]. There seems to be an inconsistency between the same effects triggered by the opioid antagonist or agonist in the two mentioned studies, however in the first case, the entire opioid system has been blocked, while in the second one, only one type of opioid receptor activity was manipulated, leaving the rest opioid receptor types unoccupied. The latter research group has also noticed that bladder cancer patients, receiving non-opioid, anesthetic drug ropivacaine ((2S)-N-(2,6-dimethylphenyl)-1-propyl-2-piperidinecarboxamide hydrochloride) during and after surgery with very limited opioid addition, had significantly decreased number of CTCs, particularly CTCs of mesenchymal type (M-CTC class), than E-CTC (epithelial CTC) and CTC clusters as compared to patients receiving systemic opioids (sufentanil and remifentanil) during perioperative period. Such opioid-sparing strategies in clinical oncology [73,74,75,76] allow to treat not only pain, but also to reduce opioid side effects, including EMT-driven cancer progression.

### 2.8. Oesophageal Cancer

Oesophageal cancer is a burden for over half a million people, as the latest global statistics show. For these patients, researchers also seem to have some positive information. In a study with the use of oesophageal cancer cell cultures (Table 2), treatment with sufentanil inhibited EMT via suppression of nuclear NF-κB and SNAIL levels [77]. On the contrary, morphine had an unfavourable effect on the same type of cancer, contributing to a decrease in E-cadherin and increase in vimentin, SLUG, and SNAIL levels, followed by enhanced migratory ability [78] (Table 2). The authors postulated the results were likely AMP—activated protein kinase (AMPK)-dependent, but MOR-independent, since naloxone failed to restore studied protein expression observed before treatment, as AMPK inhibitor did. Establishing opioid receptors levels in used cell line and testing more opioid antagonists possibly could be supportive in further seeking possible reasons for opioid antagonism failure. At least such suggestions emerge from a study on human buccal cancer TR146 cells, demonstrating greater MOR and DOR expression levels and to a lesser extent the KOR type. When these cells were pretreated to naloxone or the specific DOR antagonist naltrindole, but not selective KOR or MOR antagonist, they displayed more profound inhibition of migratory activity after subsequent morphine treatment, which suggested a DOR-mediated effect [79]. Furthermore, morphine has been found to act via alternative, opioid receptor-independent mechanisms. It has been evidenced that morphine was also able to transactivate EGFRs [80] or bind to toll-like receptor 4 (TLR4) [81].

### 2.9. Pancreatic Cancer

Worldwide incidence and mortality from pancreatic cancer are at similar level as in case of oesophageal malignancy. Recent investigations testing opioids that would draw back EMT in the outcome of pancreatic cancer or at least allow to identify novel mechanisms and therapeutic targets provided some new information. In pancreatic ductal adenocarcinoma, MOR has been found to colocalize as a functional heterodimer with somatostatin receptors 2 (SSTR2), which was not specific for normal, pancreatic cells, as confirmed by the co-immunoprecipitation procedure. Dermorphin- and L-054,264-specific agonists for MOR and SSTR2, respectively, when used separately, caused downstream EGFR phosphorylation only in PANC-1 cell, but not normal cells, with a more pronounced effect upon opioid agonist administration and no observable effect if the two compounds had been given (Table 2). However, only combination of the two ligands affected significantly the expression of classical EMT markers (vimentin, MMP9, N-, and E-cadherin) and only in pancreatic cancer cells, but not normal counterparts, towards a more aggressive phenotype, preceded by sustained phosphorylation of ERK1/2 [82]. An opposite effect demonstrated fentanyl in human pancreatic cancer SW1990 cells. The drugdose-dependently decreased α-SMA and vimentin but increased E-cadherin levels upon 48 h incubation. Interestingly, along with changes in EMT markers expression, key mediators of MAPK pathway: p38, ERK1/2, and JNK were downregulated at fentanyl doses greater than 2 ng/mL, but slightly upregulated at 0.5 ng/mL, as compared to the control [83], (Table 2). Studies mentioned above bring two additional, valuable suggestions for further research and drug development in this field. The first one concerns increased therapeutic efficacy of combined treatment including more than one molecular target; the second one encourages for examination of an extended dose range of opioids, despite their known properties, mostly being tumour-promoting.

### 2.10. Ovarian Cancer

The next suggestion on opioid use in cancer emerges from fentanyl effects on ovarian cancer. Human ovarian SK-OV-3 cells treated with the opioid demonstrated a concentration-dependent increase in vimentin, SNAIL, and SLUG expression and a decrease in claudin-1, along with enhanced phosphorylation and, thus, activation of EGFR and downstream ERK, p-90RSK, and AKT molecules (Table 2). Moreover, naloxone failed to influence p-EGFR level, though it reversed fentanyl-mediated phosphorylation of EGFR and reduced the migratory ability of ovarian cancer cells [84]. The same opioid may bring a completely different effect, beneficial or adverse, dependently on the cell line, as in the mentioned studies on fentanyl in ovarian and pancreatic cancer cell cultures.

### 2.11. Head and Neck Cancer

Head and neck cancer is driven mostly by the oropharyngeal type [85], and the laryngeal-associated malignancies are mentioned after 19th position in the GLOBOCAN 2022 statistics [37]. The opioid receptor antagonist methylnaltrexone had an EMT-preventive effect in human head and neck squamous cell carcinoma FaDu cells, including a reduction in SNAIL but not TWIST expression upon at least 48 h exposure, and a similar effect was achieved by opioid receptor µ1 gene (*OPRM1*) knockdown [86], (Table 2).

Summarizing, opioid system has an impact on tumour progression involving EMT (Table 2). Opioid drugs may demonstrate an opposite action, dependent on cancer histological type, grade, or treatment regimen. However, the data gathered so far imply a relatively consistent EMT-promoting profile of morphine and fentanyl, while sufentanil or naltrexone seem to counteract the undesirable transition of cancer cells towards a mesenchymal and, therefore, more malignant state. Current results from drug repurposing analyses encourage for further studies on naldemedine and naltrexone as promising anti-EMT agents.

**Table 2:** Summary of the effects of opioids on EMT in cancer.

Opioid	Opioid Dose /Treatment Period *	Type of Cancer /Cell Line	↑ EMT Stimulation ↓ EMT Inhibition **
Opioid receptors agonists			
β-endorphin	Transplanted β-endorphin-producing neurons/10 weeks (in vivo)	N-methyl-N-nitrosourea-induced breast cancer [56]	↓
Morphine	1–100 nM/96 h	human NSCLC H358 cell line [41]	↑
	0.5–10 µM/24 h	human esophageal cancer KYAE-1 cell line [78]	↑
	0.1–10 µM/L/24 h	human bladder carcinoma T24 cell line	↑
		human bladder 5637 cell line	↑
		mouse bladder cancer MB49 cell line [73]	↑
	10 mg/kg/day/14 days (in vivo)	mouse bladder cancer MB49 cell line [73]	↑
	10 µM/48 h	human lung cancer A549 cell line; mouse Lewis lung cancer cell line [48]	↑
Oxycodone	0.25–1 mM	human breast cancer SKBR3 cell line [63]	↓
Tramadol	0.05–1.5 mg/mL	human breast MDA-MB-231 and MCF-7 cell lines [55]	↓
Fentanyl	1–100 nM/96 h	human lung cancer H358 cell line [41]	↑
	0.01–0.1 µM/48 h	human breast cancer MCF-7 cell line [87]	↑
	0.01–0.1 µM/48 h	human breast cancer MDA-MB-231 cell line [87]	↑
	0.02 mg/kg/day/3 weeks (in vivo)	human breast cancer MCF-7 cell line [87]	↑
	100–400 nM/24 h	human ovarian cancer SK-OV-3 cell line [84]	↑
	0.5–5 ng/mL/48 h	human pancreatic SW1990 cancer cell line [83]	↓
Sufentanil	1–10 μmol/L/24 h	human esophageal cancer Eca-109 and CaES-17 cell lines [77]	↓
	20–80 nM/24 h	human breast cancer MDA-MB-231 and BT549 cell lines [59]	↓
	1 nM/24 h	human breast cancer BT474 cell line [60]	↓
	2 nM/24 h	human lung cancer H460 and H1299 cells lines [42]	↓
Dermorphin	10 nM/24 h	human pancreatic cancer PANC-1 cell line and normal pancreatic cells [82]	-
D-Ala 2, D-Leu 5]-Enkephalin (DADLE)	1 μM/5 min–72 ha	human breast cancer MCF-7 and T47D cell lines [51]	↑
	1 μM/5 min–72 ha	human breast cancer MDA-MB-231 cell line [51]	-
Nalbuphine	100 µM/48 h	human breast cancer MDA-MB-231, MCF-7 and SKBR-3 cell lines [58]	↓
	2 mg/kg/day/25 days (in vivo)	human breast cancer MDA-MB-231 [58]	↓
Methionine-enkephalin (MENK)	6 mg/mL/24–48 h	human lung cancer A549 cell line; human lung cancer H1975 cells transfected with negative control (NC) siRNA vs. the same line treated with MENK [45]	↓
Opioid receptors antagonists			
Naltrexone	1.26 mg/mL/48 h	human cervical cancer HeLa cells transfected with an empty lentiviral vector vs. the same cell line treated with naltrexone [71]	↓
	10 mg/kg/4 weeks (In vivo)	human breast cancer MDA-MB-231 cell line [61]	↓
	1–100 µM/24 h	human bladder cancer T24 cell line; mouse bladder cancer MB49 cell line [72]	↑
Methylnaltrexone	100 nM/48 h	human head and neck squamous cell carcinoma FauDu cell line [86]	↓

* treatment regimens refer to in vitro studies, if not specified otherwise. ** tendency means opioid-induced changes in some specific markers of EMT towards EMT inhibition (↓)/stimulation (↑). “-“ not clearly specified.

## 3. Opioid, EMT, and Tissue Fibrosis

Tissue fibrosis may occur in various organs and usually appears as non-cancerous lesions; however, some features can be found in the course of cancer disease. Pulmonary fibrosis has its roots in extensive, proliferative activity of fibroblasts, along with abnormal accumulation of extracellular matrix proteins, including collagen, which lead to irreversible changes in the lungs. Some reports emphasize the role of viral infection in the development of such aberrant tissue structure. Research on archival lung samples collected from macaques infected with the SIVmac251 virus and/or exposed to long-term treatment with morphine revealed a significant increase in TGF-β1, N-cadherin, and vimentin and a decline in E-cadherin and claudin-5 levels in tissue specimens from animals with either developed opioid dependency, or those that were SIV-positive, as compared to tissue specimens from naïve macaques; however, the greatest changes were observed in animals influenced by both factors [88].

Likewise, opioid involvement in EMT-associated changes has also been uncovered in renal fibrosis as sequelae of hyperglycemia and diabetes. It is considered that kidney fibrosis results from accumulation of matrix-producing myofibroblasts. One hypothesis on the origin of these mesenchymal cells indicates renal tubular epithelial cells undergoing EMT as a primary source [89]. It has been reported that stimulation of DOR may counteract the TGF-β1-promoting effect on EMT during renal fibrogenesis. In one study, the specific DOR agonist UFP-512 revealed a capacity to suppress TGF-β1-induced morphological changes and migration of rat kidney NRK 52E cells, abundantly expressing DOR. Moreover, DOR activation reduced the Tgf-β-mediated increase in fibronectin and α-Smabut also restored TGF-β-stimulated E-cadherin loss and displayed a tendency to reverse Snail increase; however, the latter observation was statistically insignificant. The DOR agonist also markedly decreased phosphorylation of Smad3, p38, and Akt in Tgf-β-treated kidney cells; however, these effects of the DOR agonist were uncovered only upon prior, or combined use with the Tgf-β cytokine, while using it alone did not affect the activity of any mentioned proteins [90].

## 4. Opioids, EMT, and Tissue Repair

In the wound healing process, an important role has been assigned to TGF-β, including its β2 isoform. Interestingly, stimulation of MOR by β-endorphin supports this cytokine release in cultured keratinocytes. MORs have been evidenced to be downregulated in the keratinocytes of the margin of chronic wounds as compared to the acute wounds. Opioids also enhance collagen deposition. On the other hand, opioids may impair wound closure by suppressing myofibroblasts recruitment. Patients with chronic wounds frequently experience associated pain, forcing them to use opioids, which were demonstrated to contribute to the delayed healing [91]. DOR-deficient mice displayed difficulties in wound repair [92]. DOR receptors are involved in desmosome integrity via regulation of protein composition. Human keratinocyte N/TERT-1 cells overexpressing DOR had β-catenin redistributed to cytosol with its depletion at cell–cell contact sites and displayed DOR-mediated activation of protein kinase C (PKC) signalling, which enhanced migratory ability, reflected by increased easiness to form actin-rich protrusions. When exposed to MENK, N/TERT-1 cells demonstrated downregulation of integrin β1 in cell–cell margins and decreased desmoglein (DSG) 1 and 4, while the DOR antagonist naltrindole increased DSG1 levels and integrin distribution in these cells [93]. Corneal epithelium alteration is a common comorbidity in diabetic patients, resulting in vision loss. Surgical removal of abnormal tissue restores vision clarity; however, later re-epithelialization is linked with some difficulties. Interestingly, a high level of [Met^5^]-enkephalin in blood has been observed in diabetic patients, as well as mouse model of genetic diabetes. In rats with streptozotocin-induced insulin-dependent diabetes, treatment with naltrexone after abrasion of corneas accelerated wound healing. Remarkably, naltrexone enhanced re-epithelialization in non-diabetic animals [94] and wound healing upon topical application in diabetic rats [95].

## 5. Future Directions for Development of Anti-Pain Strategies in Cancer, Considering Their Effect on EMT, and Conclusions

Up to date results from research included in this review seem to testify that opioid agonists rather activate EMT than prevent the transition. Although systemic use of opioids for pain treatment appears to be inevitable, particularly in end of life palliative care when improvement of well-being is of higher priority than cancer disease therapy due to exhaustion of all curative options, some opportunities still remain for most patients, allowing alleviation of pain and preventing further worsening of their illness.

During surgical resection of some primary tumours, where it is possible, the use of non-opioid analgesics, or reduction in their dose, appears to be a reasonable approach. Furthermore, regional, minimal anesthesia instead of systemic sedation applied before, e.g., brain tumour removal, may reduce opioid side effects. However, considering malignant cancer types of the central nervous system as gliomas, awake craniotomy may not bring the expected benefits for improving patient prognosis [96], and it seems that the clinical advantages of local anesthesia are restricted to selected types of malignancies [97]. Unfortunately, there are some premises for the notion that non-opioid substances administered during anesthesia as sedatives, muscle relaxants, or non-opioid analgesics may also induce EMT [98,99,100]. On the other hand, as in case of opioids, non-opioids drugs given during surgery and peri-operative period have also been demonstrated to attenuate the transition of cancer cells to a more invasive phenotype [101,102,103].

Current guidelines on opioid-sparing pain management strategies have been developed to avoid opioid side effects as dependency, immunosuppression, or constipation; however, the recommendations could be adopted in order to reduce risk of tumour recurrence [104,105,106].

One of the promising painkillers, buprenorphine, being a partial MOR agonist and KOR antagonist, is recommended alone, or in combination with naloxone, in opioid-naïve cancer patients for treatment of chronic and stable pain. The drug is more safe even in patients with impaired renal functions, and its administration is associated with less risk of hyperalgesia, addiction, respiratory depression, effects on sex hormones, or withdrawal symptoms [107,108,109,110]. Importantly, the buprenorphine effect on EMT has not been determined yet, and hopefully this gap in information will be soon filled. Subsequently, other analgesic and anesthetic drugs with an unknown “EMT profile”, both old and that are to be developed and administered to cancer patients, shall be evaluated towards their pro-/anti-EMT potency.

Chronic and severe pain accompanying advanced stages of cancer, resulting from bone metastases, besides opioids, is commonly treated with bisphosphonates (e.g., osteoclast inhibitor zoledronic acid). The drug prevents bone-related complications in the course of cancer diseases such as hypercalcemia, fractures, and associated pain. This medicine has been confirmed to provide some pain relief from bone metastases; however, it is not recommended for first-line treatment of pain due to a lack of immediate effect [111]. On the other hand, zoledronic acid seem to be promising, considering its effect on EMT [112,113,114]. Undoubtedly, further evidence from pre-clinical and clinical research is required for justification of other non-opioid anti-pain medicine use in off-label indications as protection against EMT. In addition, approved drug repurposing studies, which would target EMT signatures, represent another encouraging research direction, apart from developing new ones. Moreover, development of new formulations of pharmaceuticals for delivery through distinct routes locally (buccal, transdermal, or nasal) in order to reduce the systemic effect, particularly in patients with metastatic disease, is next, and it is a good alternative, worth exploration. Finally, combined therapies with opioid antagonists (showing a therapeutic effect preventing EMT, such as naltrexone) allow for a decrease in the dose of the opioid agonist where possible, or other opioid-sparing techniques, such as peripheral nerve block [115] and seems to be worthy of recommendation. Undoubtedly, more data on analgesics and anesthetics shall be collected to define more precise and personalized suggestions for pain control, which simultaneously would improve cancer disease outcome and patient prognosis.

## Figures and Tables

**Table 1:** EMT-related markers found in studies involving opioids.

EMT-Related Genes/Signatures *	↑ Upregulation/↓ Downregulation During EMT Process **
E-cadherin	↓
N-cadherin	↑
Vimentin	↑
Fibronectin	↑
α-SMA	↑
ZO-1	↓
Claudin-1	↓
MMP2/MMP9	↑
ZEB1/ZEB2	↑
TWIST1	↑
SNAIL (SNAI1)	↑
SLUG (SNAI2)	↑
TGF-β	↑
SMAD	↑
ERK	↑
p38 MAPK	↑
PI3K	↑
AKT	↑
WNT	↑
EGFR	↑
STAT3	↑
FGFRIIIc	↑
FGFRIIIb	↓

* EMT markers/related genes found in studies involving opioids. ** regulation of gene expression specific for EMT in general [19,33,38,39], not under influence of particular opioid substances.

## Abbreviations

AKT	serine/threonine protein kinase encoded by the oncogene in the transforming retrovirus isolated from the thymoma cell line AKT-8, which is derived from the Stock A Strain k AKR mouse; also known as protein kinase B (PKB)
AMPK	adenosine monophosphate (AMP)—activated protein kinase
α-SMA	α-smooth muscle actin
β2AR	β2 adrenergic receptor
CD36	cluster of differentiation 36
CTC	circulating tumour cell
DADLE	[D-Ala^2^, D-Leu^5^ ]-enkephalin
DAMGO	[D-Ala^2^, MePhe^4^, Gly(ol)^5^]enkephalin
DOR	δ opioid receptor
DSG1	desmoglein 1
E-cadherin	epithelial cadherin
ECM	extracellular matrix
EGFR	epidermal growth factor (EGF) receptor
EMT	epithelial to mesenchymal transition
ERK	extracellular signal-regulated protein kinase
FDA	U.S. Food and Drug Administration
FGF	fibroblast growth factor
GRB2	growth factor receptor-bound protein 2
HCC	hepatocellular carcinoma
HER	human epidermal growth factor receptor
IGF	insulin-like growth factor
IKBα	NF-κB inhibitor α
IR	insulin receptor
ITGB1	integrin β1
JAK	Janus kinase
JNK	c-Jun N-terminal kinase
KOR	κ opioid receptor
MAPK	mitogen-activated protein kinase
MENK	methionine-enkephalin
MET	mesenchymal to epithelial transition
MMP	metalloproteinase
MOR	µ opioid receptor
mTOR	mammalian target of rapamycin (protein kinase)
MYC	gene first identified as transforming sequence of avian MC29 virus causing myeloid neoplasma in chickens
N-cadherin	neural cadherin
NTX	naltrexone
NF-κB	nuclear factor κB
NOTCH	neurogenic locus notch homolog protein
OGFR	opioid growth factor (OGF) receptor
*OPRM1*	opioid receptor µ1 gene
PDGFR	platelet-derived growth factor receptor
PI3K	phosphatidylinositol 3-kinase
SLUG	snail family transcriptional repressor 2, also known as SNAI2
SMAD	family of proteins related to the mediator of *decapentaplegic* (*dpp*) signaling, *mothers against dpp* (*Mad*), in *Drosophila* and to the *Sma* genes from *Caenorhabditis elegans*
SNAIL	snail family transcriptional repressor 1, also known as SNAI1
SRC	Rous sarcoma virus *Src* gene (*v-Src*), oncogene SRC first identified in the RSV virus; non-receptor tyrosine kinase
SSTR2	somatostatin receptor 2
STAT	signal transducer and activator of transcription
TAMs	tumour-associated macrophages
TGF-β	transforming growth factor β
TNF-α	tumour necrosis factor α
TLR4	Toll-like receptor 4
TWIST	twist family a basic helix-loop-helix (bHLH) transcription factor
WNT	gene originally derived from integrase-1 in mouse breast cancer and the wingless gene of *Drosophila*; because of the two genes functional similarity, the terms were combined as the *Wnt* gene
ZEB	zinc finger E-box binding homeobox
ZO	zonula occludens
